# Benign serous ovarian tumour: a redefining moment?

**DOI:** 10.1186/1897-4287-10-S2-A83

**Published:** 2012-04-12

**Authors:** Sally Hunter, Kylie Gorringe, Michael Anglesio, Raghwa Sharma, Blake Gilks, Anna deFazio, David Huntsman, Ian Campbell

**Affiliations:** 1 Peter MacCallum Cancer Centre, Melbourne, VIC, Australia; 2 The Center for Translational and Applied Genomics (CTAG) at the British Columbia (BC) Cancer Agency, Vancouver, BC, Canada; 3 Dept of Pathology, Vancouver General Hospital, BC, Canada; 4 Department of Gynaecological Oncology, Westmead Hospital, Westmead, NSW, Australia; 5 Westmead Institute for Cancer Research, University of Sydney at Westmead Millennium Institute, Westmead Hospital, Westmead, VIC, Australia

## 

While the paradigm that malignancies arise through a stepwise progression from benign precursors has been established for many malignancies, it remains unclear if this holds true for ovarian cancer. Serous ovarian carcinomas are the predominant clinically important subtype and it has been widely believed that some or all of these arise from precursors derived from the ovarian surface epithelium, such as inclusion cysts or serous benign and borderline tumours. Despite the co-occurrence of benign, borderline and low grade carcinoma epithelial components, direct molecular evidence for the benign lesions as precursors is limited. This study aimed to perform high resolution copy number analysis using a series of benign serous ovarian tumours to identify any underlying genomic changes indicative of early events in tumourigenesis, which could assist in determining if these lesions represent precursors to some invasive serous ovarian carcinomas. This is the first ultra-high resolution copy number analysis of benign serous tumours of the ovary.

High resolution copy number analysis was performed on tumour epithelial and fibroblast DNA using the Affymetrix OncoScan and SNP6.0 array platforms. Copy number aberrations (CNAs) were detected in the epithelium of only 5.6% (2/35) of serous cystadenomas and cystadenofibromas. Unexpectedly, CNAs were detected in the tumour fibroblasts in 36% (14/39) of cases, including gain of chromosome 12 in 10 cases. No *KRAS* or *BRAF* mutations were detectable in either component of the benign serous tumours. Chromosome 12 trisomy has been previously identified in pure fibromas, supporting the concept that a significant proportion of benign serous tumours are in fact primary fibromas with an associated cystic mass. This study therefore provides a novel perspective on the development of these tumours.

